# Factors that shape the elevational patterns of plant diversity in the Yatsugatake Mountains, Japan

**DOI:** 10.1002/ece3.7397

**Published:** 2021-03-17

**Authors:** Yoshitaka Oishi

**Affiliations:** ^1^ Center for Arts and Sciences Fukui Prefectural University Fukui Japan

**Keywords:** climatic factor, elevational pattern, mass effect, mid‐domain effect, plant–plant interaction, Rapoport's elevational rule

## Abstract

Elevation is involved in determining plant diversity in montane ecosystems. This study examined whether the distribution of plants in the Yatsugatake Mountains, central Japan, substantiated hypotheses associated with an elevational diversity gradient. Species richness of trees, shrubs, herbs, ferns, and bryophytes was investigated in study plots established at 200‐m elevational intervals from 1,800 to 2,800 m. The changes in plant diversity (alpha and beta diversities, plant functional types, and elevational ranges) with elevation were analyzed in relation to climatic factors and elevational diversity gradient hypotheses, that is, mass effect, mid‐domain effect, and Rapoport's elevational rule. In addition, the elevational patterns of dominance of plant functional types were also analyzed. A comparison of alpha and beta diversities revealed that different plant groups responded variably to elevation; the alpha diversity of trees and ferns decreased, that of herbs increased, whereas the alpha diversity of shrubs and bryophytes showed a U‐shaped relationship and a hump‐shaped pattern. The beta diversity of shrubs, herbs, and bryophytes increased above the subalpine–alpine ecotone. In accordance with these changes, the dominance of evergreen shrubs and graminoids increased above this ecotone, whereas that of evergreen trees and liverworts decreased. None of the plant groups showed a wide elevational range at higher elevations. These elevational patterns of plant groups were explained by climatic factors, and not by elevational diversity gradient hypotheses. Of note, the changes in the dominance of plant groups with elevation can be attributed to plant–plant interactions via competition for light and the changes in physical habitat. These interactions could alter the elevational diversity gradient shaped by climatic factors.

## Graphical abstract

This study examined whether the distribution of plants in the Yatsugatake Mountains, central Japan, substantiated hypotheses associated with an elevational diversity gradient. The elevational patterns of plant diversity were explained by climate and plant–plant interactions, and not by hypotheses for elevational diversity distribution.

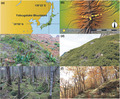

## 1 INTRODUCTION

Elevation is a key factor that determines the patterns of plant diversity in mountainous areas (Jiang et al., 2018; Körner, 2007; Lee et al., 2013; Lee & La Roi, 1979). The patterns of plant diversity are often explained by several hypotheses for elevational diversity gradient such as the mass effect, mid‐domain effect, and Rapoport's elevational rule. According to the mass effect hypothesis, a species disperses from source areas occupied by larger populations to the adjacent sink areas; however, the dispersed species cannot maintain viable populations in the sink areas (Grytnes et al., 2006; Shmida & Wilson, 1985). These temporal populations increase species richness around ecotones where two different communities meet. Unlike this hypothesis, the mid‐domain effect hypothesis assumes a random distribution of species within a geometrically constrained domain such as that between the summit and base of a mountain. Consequently, species richness peaks in the middle of the domain because of the increasing overlap of species distribution toward the center (Colwell & Lees, 2000; Colwell et al., 2004). The Rapoport's elevational rule postulates that species at higher elevations can withstand a broad range of climatic conditions across a high range of elevations (Stevens, 1992).

However, explanation of plant elevational patterns using these hypotheses is often debatable. This is because plant groups show disparate responses to elevation, and even plants within a group present dissimilar elevational distribution in different areas (Bhattarai & Vetaas, 2003; Bruun et al., 2006; Grytnes et al., 2006; Miyajima et al., 2007; Sánchez‐González & López‐Mata, 2005). Hence, the applicability and limitations of these hypotheses for elevational diversity gradient should be further explored through careful examination of relationships between plant distribution and elevation.

Such an examination in mountainous areas in Japan is of interest because of the characteristic vegetation at higher elevations. These areas experience strong winds and heavy snowfall owing to the influence of jet streams and winter monsoons (Manabe, 1957; Riehl, 1962; Ueda et al., 2015). The severe climatic conditions enable a single Japanese stone pine tree species (*Pinus pumila* Regel) to become the dominant vegetation above the subalpine–alpine ecotone owing to its resistance to wind and snow (Okitsu, 1984). The dominance of *P. pumila* could alter the elevational patterns of plant diversity from those predicted based on the above‐mentioned hypotheses (mass effect, mid‐domain effect, and Rapoport's elevational rule). This is because the subalpine–alpine ecotone is otherwise dominated by a mixture of alpine and subalpine plants according to the mass effect. From other perspectives, the dominance of *P. pumila* could reduce the randomness of species distribution as postulated by the mid‐domain effect and hinder the dispersal of alpine species in lower elevations against the assumption based on the Rapoport's elevational rule. Hence, testing the elevational hypotheses in this region can contribute to the understanding of the applicability and limitations of these hypotheses.

In this study, the elevational distribution of multiple plant groups in the Yatsugatake Mountains, Japan, was evaluated to determine whether the hypotheses for elevational diversity gradient (i.e., the mass effect, mid‐domain effect, and Rapoport's elevational rule) could substantiate the existing elevational patterns of the plant groups. If these hypotheses could explain the elevational patterns of plants, plant diversity can exhibit the following changes with elevation.

Hypothesis 1 If the elevational patterns of plants follow the mass effect hypothesis, subalpine and alpine species should coexist in the subalpine‐alpine ecotone. As a result, the alpha diversity will be the highest around this ecotone. Furthermore, beta diversity can be the highest in this ecotone owing to the coexistence of subalpine and alpine plants.

Hypothesis 2 If the mid‐domain effect hypothesis is applied to the elevational patterns, the overlap of species distribution will increase toward the center between the summit and base of a mountain. Consequently, the alpha diversity will be the highest around the middle elevations.

Hypothesis 3 If the distribution of plant species can be explained by the Rapoport's elevational rule, alpine species will exhibit larger elevational ranges than species at lower elevations. Hence, the alpha diversity will be relatively high at lower elevations where both alpine and lowland species coexist.

Hypothesis 4 If the three elevational hypotheses do not apply to the observed patterns of plant distribution, other factors, such as climate and plant–plant interactions, will largely determine their distribution. Specifically, the influence of plant–plant interactions may be presented in relationships between the ecological traits of plant groups (plant functional types).

## 2 MATERIALS AND METHODS

### 2.1 Study site

**FIGURE 1:**
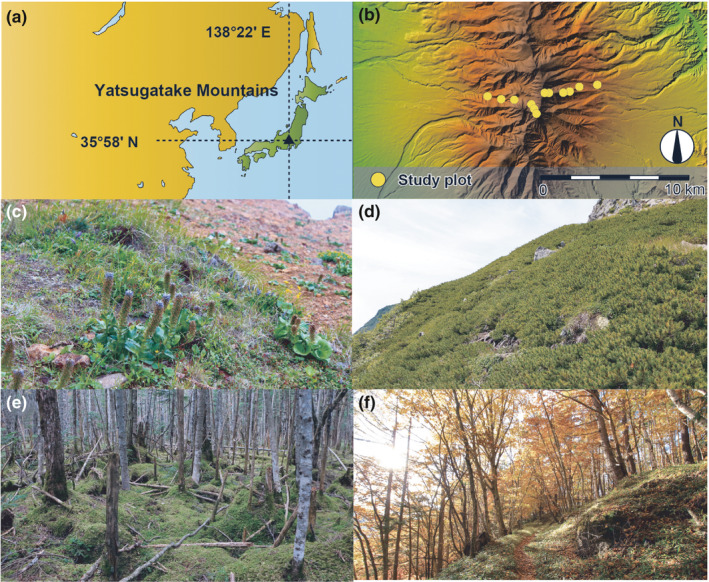
Location of the study site and plots, and the major vegetation types in the Yatsugatake Mountains, central Japan: (a) location of the Yatsugatake Mountains, (b) location of the study plots, (c) alpine meadow, (d) alpine dwarf pine scrub (*Pinus pumila*), (e) subalpine conifer forest, and (f) temperate deciduous forest. A part of this figure was adapted from Oishi (2019)

This study was conducted in the Yatsugatake Mountains (Figure 1). The highest peak in these mountains is the 2,899 m summit of Mount Akadake (35°58′15″N, 138°22′12″E). The vegetation in the Yatsugatake Mountains is largely classified into the following four types: temperate deciduous forest dominated by *Quercus crispula* and *Larix kaempferi* below ~1,800 m; subalpine conifer forest dominated by *Tsuga diversifolia*, *Abies veitchii*, and *Abies mariesii* f. *hayachinensis* at ~1,800–2,600 m; alpine dwarf pine scrub dominated by *P. pumila* at ~2,600–2,800 m; and alpine meadow with turf‐rock vegetation at ~2,800–2,900 m. The highest and lowest mean temperatures in the nearest weather station at 1,350 m are 19.2°C in August and −5.3°C in January, and the mean precipitation is 1,439.9 mm/year (Japan Meteorological Agency, 2020).

### 2.2 Field survey

Twelve 10 × 10 m study plots were established at 200‐m elevational intervals from 1,800 to 2,800 m (i.e., 1,800, 2,000, 2,200, 2,400, 2,600, and 2,800 m) along two trails, extending from the east to the west of the Yatsugatake Mountains (Figure 1b). This plot size was determined using the minimal area method in areas with uniform microtopography. The plant species in each plot were recorded based on the taxonomic groups: trees, shrubs, herbs, ferns, and bryophytes. Shrubs are woody plant species of height less than 2.0 m. Ferns and bryophytes included both epiphytic and terrestrial species. Epiphytic species were recorded up to 2.0 m from the ground.

To examine the influence of climate on plant diversity, temperature and relative humidity (RH) were measured in each plot at ~5 cm above the ground at 4 hr intervals throughout the year, using HOBO U23 Pro v2 temperature/relative humidity data loggers (Onset Computer Corporation). To reduce the influence of sunlight on the measurements, the data loggers were covered with a CO‐RS1 solar radiation shield (Onset Computer Corporation). Using the temperature and RH data, the following climatic variables were calculated: the mean annual temperature (Temp_ann_), mean temperature during the growing season (Temp_grow_), mean temperature during the snow season (Temp_snow_), mean annual RH (RH_ann_), mean RH during the growing season (RH_grow_), mean RH during the snow season (RH_snow_), and duration of the snow season (snow cover). A series of snow‐covered days with over 90% RH was defined as the snow season. This definition was based on a primary experiment in which data loggers were buried under a snow pack. The remaining days were defined as the growing season. RH_snow_ was not used in the following analysis because this variable is nearly uniform according to its definition.

### 2.3 Statistical analyses

Alpha diversity, beta diversity, dominance of functional types, and elevational range of each plant group were calculated to examine the validity of the hypotheses for elevational diversity gradient. Alpha diversity was defined as the species richness of each plant group within a plot, and its correlation with elevation was fitted using generalized linear models (GLMs) with log‐link function and Poisson distribution. Models were selected as follows (model selection 1). Both linear and second‐order polynomial models were constructed, and the final model with significant coefficients and the lowest second‐order Akaike information criterion (AICc) value was selected. If a model did not have significant coefficients, then the model with the lowest AICc value was adopted.

The influence of climatic factors on alpha diversity was also analyzed using GLMs with log‐link function and Poisson distribution. The final model was selected as follows (model selection 2). Climatic factors that strongly correlated with alpha diversity (*r* > 0.5) were selected as explanatory variables. The models with different combinations of these explanatory variables were then constructed. Among these models, the final model had significant coefficients and the lowest AICc value.

Beta diversity was evaluated using the *β*_sim_ index, considering that the performance of this metric was excellent under various criteria (Koleff et al., 2003). One of the advantages of this index is that it focuses more on compositional differences between the focal and neighboring quadrats than on the differences in species richness (Koleff et al., 2003). The *β*_sim_ index was calculated between the adjacent plots as follows:

(1) $$\beta_{\text{sim}}=1‐\frac{a}{\left(\text{min}[b,c]+a\right)}$$

where *a* is the total number of species found in both plots, *b* is the number of species that occur in the first plot but not in the second plot, and *c* is the number of species that occur in the second plot but not in the first plot. The changes in beta diversity with elevation were fitted by linear models (LMs). The final model was selected according to model selection 1. The influences of climatic factors on beta diversity were analyzed using LMs. The final model was determined based on model selection 2.

The elevational patterns of plants were further discussed in relation to the changes in plant functional types. Plant functional types are groups of species that respond similarly to environmental and biotic changes (Duckworth et al., 2000). These responses were used to characterize the elevational patterns of plant groups (Bruun et al., 2006; Sánchez‐González & López‐Mata, 2005; Zhou et al., 2019). In this study, trees, shrubs, herbs, and bryophytes were classified into the following functional types: evergreen and deciduous trees, evergreen and deciduous shrubs, forbs and graminoids, and mosses and liverworts, respectively. The dominance of plant functional types was calculated as the ratio of the species richness of each functional type to the total species richness of the respective plant group in each plot. The changes in the dominance of plant functional types with elevation were fitted by LMs, and the final model was selected according to model selection 1. The relationships between the dominance of plant functional types were also examined using Pearson correlation to check the interactions among the plant groups.

To test the applicability of the mid‐domain effect to elevational distribution, the discrepancy between the observed results and those predicted based on the mid‐domain effect was determined using the null model (discrete mid‐domain effect model) (Colwell & Hurtt, 1994). The predicted mean species richness was calculated by 9,999 Monte Carlo simulations using RangeModel software version 5.0 (Colwell, 2006). The predicted values were plotted against elevation and correlated with the observed alpha diversity using Pearson's correlation.

Finally, the applicability of the Rapoport's elevational rule to elevational distribution was examined based on the elevational range of each plant group. The elevational range was defined as the mean difference between the highest and lowest elevations of all the recorded species in each plot and was calculated as follows:

(2) $$\text{Elevational range}=\frac{\sum_{i=1}^{n}\left(H_{i}‐L_{i}\right)}{n}$$

where *n* is the total number of species recorded in a plot, *H_i_* is the highest elevation where the *i*th species was recorded, and *L_i_* is the lowest elevation where the *i*th species was recorded. The highest and lowest elevations were based on elevation records in the study site, herbarium specimens (Nagoya University Museum; NUM‐Bt) collected in central Japan (Chubu, Kinki, and Kanto regions), plant diversity databases (Biodiversity Centre of Japan, 2020), and plant survey reports from the studied region (Akiyama, 1983, 1984; Hattori, 1958; Inoue, 1981; Kodama, 1971, 1972; Masuzaki & Katagiri, 2010; Takaki et al., 1970). The changes in elevational ranges with elevation were examined using LMs. This modeling was performed with the same procedure for LMs between beta diversity and elevation.

## 3 RESULTS

### 3.1 Species richness, elevational distribution, and climatic factors

Two hundred and sixty plant species (20 tree species, 17 shrubs, 49 herbs, 11 ferns, and 163 bryophytes) were recorded in the study plots. The species richness per plot (mean ± *SD*) of trees, shrubs, herbs, ferns, and bryophytes was 5.00 ± 1.81, 2.50 ± 2.32, 9.42 ± 5.14, 2.00 ± 1.86, and 41.00 ± 17.00, respectively (Appendix S1).

The changes in climatic factors with elevations are presented in Appendix S2. The Pearson's correlation analysis between climatic factors and elevation showed that elevation positively correlated with the snow cover (*r* = 0.769; *p* < 0.01) but negatively correlated with Temp_ann_ (*r* = −0.862; *p* < 0.01) and Temp_grow_ (*r* = −0.822; *p* < 0.01). In contrast, no significant correlations were observed between elevation and the RH variables (RH_ann_ and RH_grow_). These variables were the highest at the mid‐elevations, showing hump‐shaped relationships with elevation.

### 3.2 Alpha diversity

**FIGURE 2:**
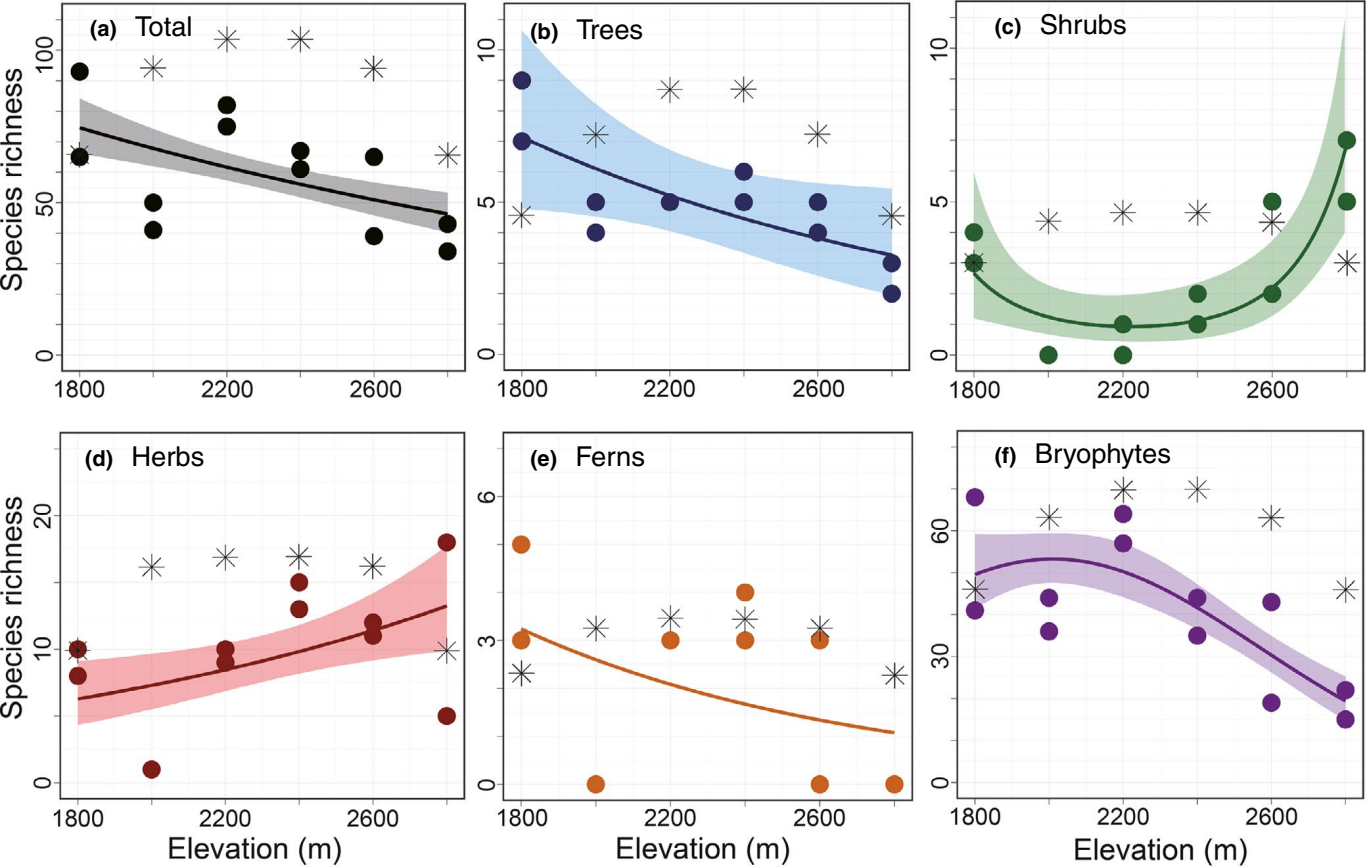
Elevational changes in the alpha diversity of plant groups in the Yatsugatake Mountains, central Japan: (a) total, (b) trees, (c) shrubs, (d) herbs, (e) ferns, and (f) bryophytes. The calculated alpha diversity based on the mid‐domain effect is denoted with an asterisk (*). To illustrate the alpha diversity, generalized linear models were used to explore the pertinent changes with elevation. When the coefficient was significant, a 95% confidence interval was calculated and colored

The alpha diversity of the plant groups varied with elevational distribution (Figure 2). All coefficients, except those for ferns, in the GLMs were significant (Appendix S3). The alpha diversity of both total plants and trees decreased with elevation (Figure 2a,b), whereas that of herbs increased (Figure 2d). The alpha diversity of bryophytes showed a hump‐shaped pattern (Figure 2f) and that of shrubs showed a U‐shaped relationship with the highest value at an elevation of 2,800 m (Figure 2c). The alpha diversity of herbs increased with elevation; however, the highest alpha diversity on an average was recorded in the 2,400‐m plot.

The results of alpha diversity and climatic factors obtained using GLMs are shown in Table 1. The variables Temp_ann_ and Temp_snow_ presented significant positive effects on the alpha diversity of trees and ferns. The alpha diversity of shrubs negatively correlated with the RH_grow_, whereas that of herbs positively correlated with snow cover and that of bryophytes positively correlated with Temp_ann_ and RH_grow_.

**TABLE 1:** Generalized linear model results between alpha diversity and climate variables

Res. var	Exp. var	Estimate	*SE*	*Z*‐value	*p*‐value	*R* ^2^
Total	Temp_ann_	0.133	4.58 × 10^−2^	2.91	<0.01	0.927
	Temp_snow_	−5.73 × 10^−2^	4.03 × 10^−2^	−1.42	0.155	
	RH_ann_	0.126	3.92 × 10^−2^	3.22	<0.01	
Trees	Temp_ann_	0.206	9.48 × 10^−2^	2.18	0.0296	0.779
	Intercept	−55.4	26.2	−2.11	0.0347	
Shrubs	RH_grow_	−0.234	6.89 × 10^−2^	−3.39	<0.01	0.686
	Intercept	22.4	6.28	3.57	<0.01	
Herbs	Snow cover	1.54 × 10^−2^	4.91 × 10^−3^	3.15	<0.01	0.592
	Intercept	−0.414	0.863	−0.480	0.632	
Ferns	Temp_snow_	0.360	0.164	2.20	0.0280	0.519
	Intercept	−97.0	44.5	−2.18	0.0294	
Bryophytes	Temp_ann_	0.113	3.51 × 10^−2^	3.20	<0.01	0.977
	RH_grow_	8.60 × 10^−2^	2.01 × 10^−2^	4.29	<0.01	
	Intercept	−4.69	1.85	−2.54	0.0111	

Abbreviations: Res. var = response variable, Exp. var = explanatory variable, *SE* = standard error, *R* ^2^ = Nagelkerke's *R* ^2^, Temp_ann_ = mean annual temperature, Temp_snow_ = mean temperature during the snow season, RH_ann_ = mean annual relative humidity, RH_grow_ = mean relative humidity during the growing season, and Snow cover = duration of snow season.

### 3.3 Mid‐domain effect

Species richness (alpha diversity) was predicted using a discrete mid‐domain effect model to examine the applicability of this hypothesis to the sampling site (Figure 2, Appendix S4). In all plant groups, the correlations between the observed species richness and those predicted based on the mid‐domain effect were not positively significant (Table 2).

**TABLE 2:** Pearson correlation coefficients between species richness and that predicted using a discrete mid‐domain effect model

	Total	Trees	Shrubs	Herbs	Ferns	Bryophytes
Cor.	0.160	−0.031	−0.738**	−0.074	0.110	0.276

Abbreviation: Cor. = Pearson correlation coefficient.

* *p* < 0.05,

** *p* < 0.01.

### 3.4 Beta diversity

**FIGURE 3:**
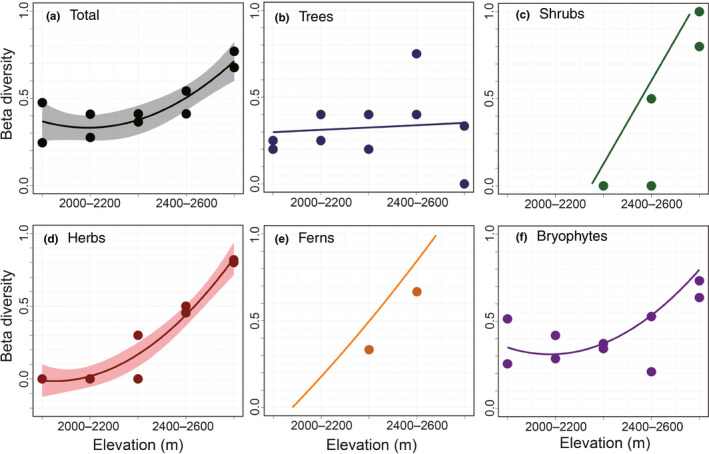
Elevational changes in the beta diversity (β_sim_ index) of plant groups in the Yatsugatake Mountains, central Japan: (a) total, (b) trees, (c) shrubs, (d) herbs, (e) ferns, and (f) bryophytes. To illustrate the beta diversity, linear models were used to explore the pertinent changes with elevation. When the coefficient was significant, a 95% confidence interval was calculated and colored

When beta diversity (based on the β_sim_ index) was plotted against elevation (Figure 3, Appendix S5), the β_sim_ index of shrubs and ferns was not calculated in several plots because of the absence of these plant groups (Figure 3c,e). The β_sim_ of shrubs and herbs substantially increased and that of bryophytes slightly increased above the subalpine–alpine ecotone (Figure 3c,d,f).

The results of LMs between the β_sim_ index and environmental factors are presented in Table 3. Similar to the results of alpha diversity, snow cover and RH_grow_ exerted a significant influence on the beta diversity of herbs and bryophytes. LMs with significant coefficients were not constructed for the beta diversity of trees and ferns.

**TABLE 3:** Linear model results between beta diversity and climate variables

Res. var	Exp. var	Estimate	*SE*	*t*‐value	*p*‐value	*R* ^2^
Total	RH_ann_	−9.21 × 10^−2^	2.67 × 10^−2^	−3.45	0.011	0.666
	Snow cover	6.09 × 10^−3^	1.82 × 10^−3^	3.34	0.012	
	Intercept	8.27	2.54	3.26	0.014	
Trees	RH_ann_	7.56 × 10^−2^	5.07 × 10^−2^	1.49	0.174	0.217
	Intercept	−6.95	4.88	−1.43	0.192	
Shrubs	Tem_ann_	−0.496	0.126	−3.94	0.029	0.784
	Intercept	1.70	0.329	5.17	0.014	
Herbs	Temp_grow_	−9.44 × 10^−2^	6.78 × 10^−2^	−1.39	0.213	0.941
	RH_ann_	−0.191	2.34 × 10^−2^	−8.15	<0.01	
	Snow cover	1.29 × 10^−2^	2.62 × 10^−3^	4.91	<0.01	
	Intercept	17.2	2.55	6.74	<0.01	
Ferns	RH_grow_	−8.71 × 10^−2^	5.32 × 10^−2^	−1.64	0.349	0.456
	Intercept	8.69	5.04	1.72	0.335	
Bryophytes	RH_grow_	−5.32 × 10^−2^	2.11 × 10^−2^	−2.52	<0.01	0.374
	Intercept	5.39	1.96	2.74	<0.01	

Abbreviations: Res. var = response variable, Exp. var = explanatory variable, *SE* = standard error, *R* ^2^ = adjusted *R* ^2^, Temp_ann_ = mean annual temperature, Temp_grow_ = mean temperature during the growing season, RH_ann_ = mean annual relative humidity, RH_grow_ = mean relative humidity during the growing season, and Snow cover = duration of snow season.

### 3.5 Functional types

**FIGURE 4:**
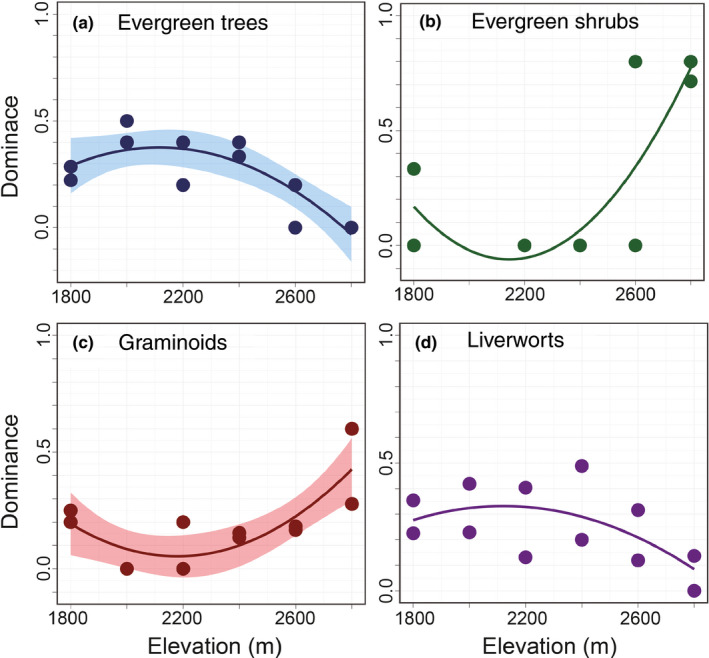
Elevational changes in the dominance of plant functional types in the Yatsugatake Mountains, central Japan: (a) evergreen trees, (b) evergreen shrubs, (c) graminoids, and (d) liverworts. To illustrate the dominance of plant functional types, linear models were used to explore the pertinent changes with elevation. When the coefficient was significant, a 95% confidence interval was calculated and colored

Elevational patterns of dominance of plant functional types are shown in Figure 4 and Appendix S6. The changes in the dominance of plant functional types were notable around 2,600–2,800 m (Figure 4). The dominance of evergreen trees and liverworts decreased in this elevation range (Figure 4a,d), whereas that of evergreen shrubs and graminoids increased (Figure 4b,c).

Regarding the relationships between functional types, the dominance of evergreen trees had a negative influence on that of evergreen shrubs and graminoids whereas it positively affected the dominance of liverworts (Table 4).

**TABLE 4:** Pearson correlation coefficients of dominance among plant functional types

	Eve. trees	Eve. shrubs	Graminoids	Liverworts
Eve. Trees	1.000	−0.899**	−0.766**	0.596*
Eve. shrubs		1.000	0.639	−0.388
Graminoids			1.000	−0.499
Liverworts				1.000

Abbreviations: Eve. trees; evergreen trees, Eve. shrubs; evergreen shrubs.

* *p* < 0.05,

** *p* < 0.01.

### 3.6 Elevational range

**FIGURE 5:**
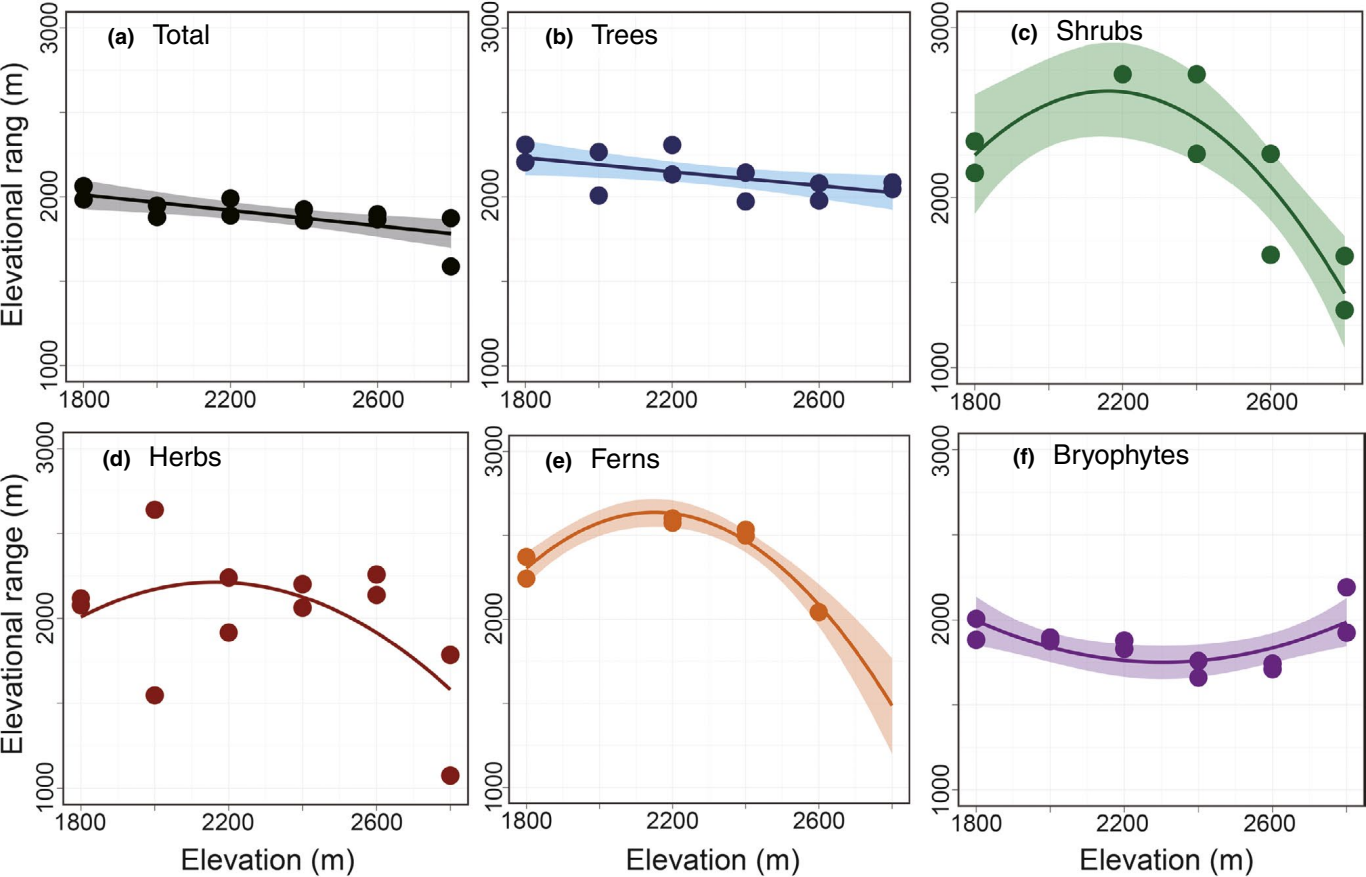
Elevational changes in the distribution range of plant groups in the Yatsugatake Mountains, central Japan: (a) total, (b) trees, (c) shrubs, (d) herbs, (e) ferns, and (f) bryophytes. To illustrate the elevational changes, linear models were used to explore the pertinent changes with elevation. When the coefficient was significant, a 95% confidence interval was calculated and colored

The mean elevational range of each plant group was calculated and plotted as a function of elevation (Figure 5, Appendix S7). None of the plant groups presented a wider elevational range at higher elevations.

## 4 DISCUSSION

In this study, elevational distribution of multiple plant groups was investigated in the Yatsugatake Mountains to determine whether the hypotheses for elevational diversity distribution could substantiate the existing elevational patterns of plant groups. The plant groups showed varied responses to the elevational gradients and climatic factors in the study site. Regarding the elevational changes in alpha diversity, only shrubs presented higher alpha diversity in the subalpine–alpine ecotone, as predicted based on the mass effect. However, none of the plant groups exhibited elevational distribution patterns corresponding to those predicted based on the mid‐domain effect or Rapoport's elevational rule. In contrast, the alpha diversity of all plant groups was explained by the climatic factors. The dominance of plant functional types correlated with each other.

### 4.1 Elevational patterns of plants

The alpha diversity of trees decreased with elevation (Figure 2b), and this elevational pattern was explained by Temp_ann_. These results are supported by the findings that the elevational range of trees can be determined by temperature through changes in their physiochemical activities (Körner, 2003). This obvious influence of Temp_ann_ on tree diversity might have led to the relatively constant changes in the beta diversity of trees compared with that of other plant groups (Figure 3b) owing to a linear decrease in Temp_ann_ with elevation.

Similar to the results for trees, the alpha diversity of ferns decreased with elevation (Figure 2e), which can be attributed to Temp_snow_ in the GLM. These results are consistent with a decrease in fern richness at higher elevation and lower temperature in central Japan (Tanaka & Sato, 2013, 2014).

In contrast to the results for the trees and ferns, the alpha diversity of herbs positively correlated with elevation (Figure 2d). Given that snow cover positively affected herb diversity in the GLM, the species richness of herbs increased with the environmental changes related to snow cover, such as soil moisture and canopy closure. Long periods of snow cover augment soil moisture (Hardy et al., 2001) essential for the proliferation of herbs in arctic–alpine habitats (Litaor et al., 2008; Nabe‐Nielsen et al., 2017; Roux et al., 2013). In addition, snow cover decreases canopy closure by suppressing tree canopy growth (Song et al., 2017), which improves light intensity and reduces litter accumulation on the forest floor. These changes in soil moisture and canopy closure could enhance herb richness by guaranteeing the photosynthesis and germination of herbs.

The alpha diversity of shrubs rapidly increased from the subalpine–alpine to the alpine zone (Figure 2c). This elevational pattern was explained by the RH_grow_ in the GLM. Considering that canopy closure reduces shrub diversity (Speziale et al., 2010) and intensifies air humidity (Jung et al., 2017), RH_grow_ may be selected in the GLM as a surrogate to represent the negative influence of canopy closure on shrub richness. Accordingly, this change in canopy closure from dense to open could have caused the rapid increase in the beta diversity of shrubs above the subalpine–alpine ecotone (Figure 3c).

Only the bryophytes showed a hump‐shaped change in alpha diversity with an increase in elevation (Figure 2f). The GLM results demonstrated that this pattern could be mainly attributed to RH_grow_. This relationship is consistent with the results of a previous study, which suggested that water stress is a major determinant of bryophyte diversity (Grau et al., 2007). The negative correlation between RH_grow_ and bryophyte beta diversity (Table 3) implies that water stress facilitates species turnover via the disappearance of drought‐sensitive species.

### 4.2 Functional types and plant–plant interactions

Here, the elevational patterns of plant diversity are further discussed in relation to plant functional types. Evergreen shrub species (*P. pumila*) have started to dominate the subalpine–alpine ecotone instead of evergreen conifer trees (Figure 4a,b). This replacement has caused a reduction in canopy closure above the ecotone, as indicated by the negative correlation between the dominance of evergreen trees and evergreen shrubs (Table 4).

In accordance with the decrease in evergreen trees, the dominance of graminoids increased but that of liverworts decreased (Figure 4c,d). These correlations can be related to improved light availability and increased drought stress owing to the change from a closed to an open canopy. Graminoids dominate open forests (Roberts & Zhu, 2002; Thomas et al., 1999) and are more resistant to drought stress than forbs (Rosbakh et al., 2017), whereas liverworts are more shade adapted (Marschall & Proctor, 2004) and are more vulnerable to drought stress than mosses (Grau et al., 2007; Jan & Wolf, 1993). These plant–plant interactions resulting from the change in canopy closure would affect the species composition, altering elevational patterns of plants influenced by climatic factors. These interactions might then facilitate species turnover around subalpine–alpine ecotone, and increase the beta diversity of shrubs, herbs, and bryophytes (Figure 3c,d,f).

### 4.3 Mass effect on elevational patterns

The validity of Hypothesis 1 is assessed based on an analysis of elevational patterns of the alpha diversity of plant groups. Among the evaluated plant groups, only shrubs showed a high alpha diversity in the vicinity of the subalpine–alpine ecotone (Figure 2), and all elevational patterns, including those of shrubs, could be explained by climatic factors and plant–plant interactions (Sections 4.1 and 4.2). Thus, the diversity of plants did not correspond to that predicted based on the mass effect. The plant–plant interactions might limit the predicted increase in species richness in the subalpine–alpine ecotone, given that the dominance of *P. pumila* inhibits the establishment of small plants by shading.

### 4.4 Mid‐domain effect and the Rapoport's elevational rule on elevational patterns

The validity of Hypotheses 2 and 3 is discussed through a comparison between the predicted and actual plant diversity. The alpha diversity of all plant groups differed from that predicted based on the mid‐domain effect hypothesis (Figure 2, Table 2). On the contrary, a higher alpha diversity of trees and bryophytes at lower elevations (Figure 2b,f) seems to validate the Rapoport's elevational rule. However, considering that none of the plant groups showed a wider elevational range in the alpine area (Figure 5), the Rapoport's elevational rule cannot be applied to the elevational patterns. Thus, both Hypotheses 2 and 3 were denied according to these results.

The lack of applicability of the mid‐domain effect and Rapoport's rule to elevational patterns can be related to the simple assumptions of these hypotheses; the former excludes any effects of gradients on the expected patterns (Colwell et al., 2004), whereas the latter fails when species distribution is affected by the interaction of several factors (Bhattarai & Vetaas, 2006; Grau et al., 2007). Specific to the study site, the climate and plant‐plant interactions at higher elevations are characterized by severe winter climates and the resultant dominance of *P. pumila* above the subalpine–alpine ecotones. These characteristics can strengthen the relationships of species distribution with climate and vegetation, and thereby increase discrepancies between the actual and predicted elevational patterns by the mid‐domain effect hypothesis and Rapoport's elevational rule.

## 5 CONCLUSIONS

In summary, the elevational patterns of plant diversity in the Yatsugatake Mountains were explained by climate and plant–plant interactions, and not by hypotheses for elevational diversity distribution (Hypothesis 1, 2, 3). Therefore, these results correspond to Hypothesis 4, indicating that the applicability of hypotheses for elevational diversity distribution largely depends on the environment and biota in the targeted study site.

Furthermore, this study emphasizes the importance of studies on elevational patterns using multiple plant groups and multiple indices of plant diversity (e.g., alpha and beta diversities, and functional types). These groups and indices are necessary to understand the characteristics of plants’ responses to elevation and their interactions that influence elevational diversity distribution.

## CONFLICT OF INTEREST

The author declares no conflicts of interest.

## AUTHOR CONTRIBUTION

**Yoshitaka Oishi:** Conceptualization (lead); data curation (lead); formal analysis (lead); funding acquisition (lead); investigation (lead); methodology (lead); project administration (lead); supervision (lead); validation (lead); visualization (lead); writing – original draft (lead); writing – review and editing (lead).

## Supporting information

Appendix S1‐S7

## Data Availability

The datasets used in this study are available in the Supporting information.
