# Supplementary material for: Factors that shape the elevational patterns of plant diversity in the Yatsugatake Mountains, Japan
Source: Ecol Evol. 2021 Mar 17;11(9):4887–97. doi: 10.1002/ece3.7397 (PMC8093696; doi:10.1002/ece3.7397)
Supplement: Supplementary file 1 — Appendix S1‐S7 [file ECE3-11-4887-s001.docx]

**Appendices**

**Appendix S1. Species richness of plant groups in the study plots**

| Plot no. | Elevation (m) | Total | Trees | Shrubs | Herbs | Ferns | Bryophytes |
| --- | --- | --- | --- | --- | --- | --- | --- |
| 1 | 1800 | 93 | 7 | 3 | 10 | 5 | 68 |
| 2 | 2000 | 50 | 5 | 0 | 1 | 0 | 44 |
| 3 | 2200 | 75 | 5 | 1 | 9 | 3 | 57 |
| 4 | 2400 | 67 | 5 | 2 | 13 | 3 | 44 |
| 5 | 2600 | 39 | 4 | 5 | 11 | 0 | 19 |
| 6 | 2800 | 43 | 3 | 7 | 18 | 0 | 15 |
| 7 | 1800 | 65 | 9 | 4 | 8 | 3 | 41 |
| 8 | 2000 | 41 | 4 | 0 | 1 | 0 | 36 |
| 9 | 2200 | 82 | 5 | 0 | 10 | 3 | 64 |
| 10 | 2400 | 61 | 6 | 1 | 15 | 4 | 35 |
| 11 | 2600 | 65 | 5 | 2 | 12 | 3 | 43 |
| 12 | 2800 | 34 | 2 | 5 | 5 | 0 | 22 |

**Appendix S2. Elevational changes in climatic variables in the Yatsugatake Mountains, central Japan**


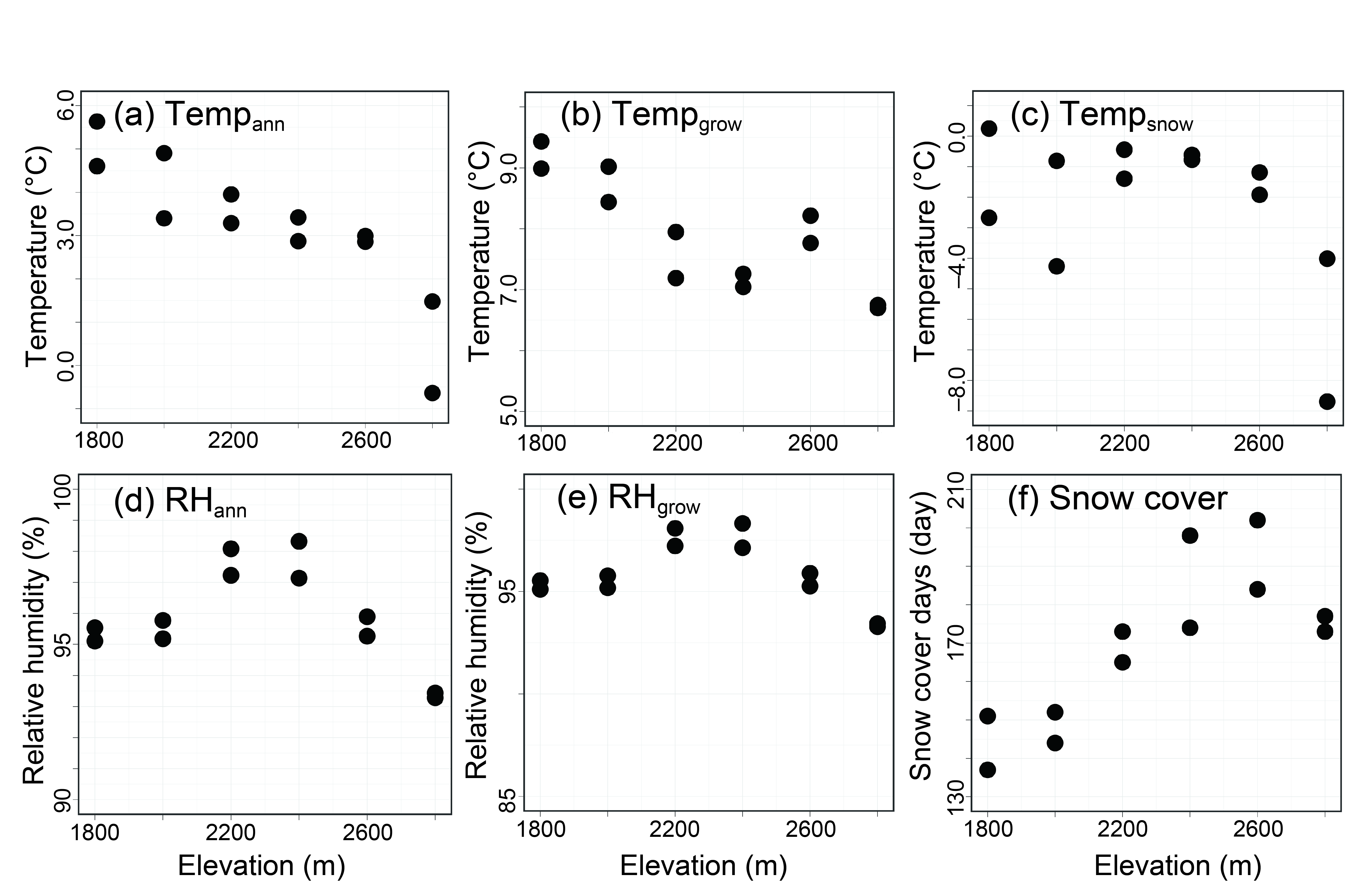


Temp_ann_ = mean annual temperature, Temp_grow_ = mean temperature during the growing season, Temp_snow_ = mean temperature during the snow season, RH_ann_ = mean annual relative humidity, RH_grow_ = mean relative humidity during the growing season, and Snow cover = duration of the snow season

**Appendix S3. Generalized linear model results between alpha diversity and elevation**

| Res. var | Exp var. | Estimate | Std. error | z−value | p-value | R^2^ |
| --- | --- | --- | --- | --- | --- | --- |
| Total | Elevation | −4.76 × 10^−4^ | 1.10 × 10^−4^ | −4.31 | < 0.01 | 0.983 |
|  | Intercept | 5.17 | 25.1 | 20.6 | < 0.01 |  |
| Trees | Elevation | −7.83 × 10^−4^ | 3.87 × 10^−4^ | −2.03 | 0.043 | 0.655 |
|  | Intercept | 3.37 | 0.864 | 3.91 | < 0.01 |  |
| Shrubs | Elevation ^2^ | 5.97 × 10^−6^ | 1.98 × 10^−6^ | 3.02 | < 0.01 | 0.829 |
|  | Elevation | −2.65 × 10^−2^ | 9.21 × 10^−3^ | −2.88 | < 0.01 |  |
|  | Intercept | 0.294 | 1.04 × 10 | 2.82 | < 0.01 |  |
| Herbs | Elevation | 7.46 × 10^−4^ | 2.81 × 10^−4^ | 2.65 | < 0.01 | 0.469 |
|  | Intercept | 49.5 | 0.495 | 0.731 | 0.465 |  |
| Ferns | Elevation | −1.10 × 10^−3^ | 6.25× 10^−4^ | −1.77 | 0.077 | 0.266 |
|  | Intercept | 7.73 × 10^−2^ | 1.37 | 2.30 | 0.021 |  |
| Bryophytes | Elevation ^2^ | −1.62 × 10^−6^ | 4.85 × 10^−7^ | −3.34 | < 0.01 | 0.983 |
|  | Elevation | 6.51 × 10^−3^ | 2.20 × 10^−3^ | 2.97 | < 0.01 |  |
|  | Intercept | −2.57 | 2.45 | −1.05 | 0.294 |  |

Res. var = response variable, Exp. var = explanatory variable, Std. error = standard error, R^2^ = Nagelkerke's R squared

**Appendix S4. Species richness predicted using discrete mid-domain effect models**

| Plot no. | Elevation (m) | Total | Trees | Shrubs | Herbs | Ferns | Bryophytes |
| --- | --- | --- | --- | --- | --- | --- | --- |
| 1 | 1800 | 65.9 | 4.6 | 3.0 | 9.9 | 2.3 | 46.0 |
| 2 | 2000 | 94.2 | 7.2 | 4.4 | 16.2 | 3.3 | 63.2 |
| 3 | 2200 | 103.6 | 8.7 | 4.6 | 16.9 | 3.5 | 69.8 |
| 4 | 2400 | 103.5 | 8.7 | 4.7 | 16.9 | 3.4 | 69.9 |
| 5 | 2600 | 94.1 | 7.2 | 4.3 | 16.2 | 3.2 | 63.1 |
| 6 | 2800 | 65.6 | 4.6 | 3.0 | 9.9 | 2.3 | 46.0 |
| 7 | 1800 | 65.9 | 4.6 | 3.0 | 9.9 | 2.3 | 46.0 |
| 8 | 2000 | 94.2 | 7.2 | 4.4 | 16.2 | 3.3 | 63.2 |
| 9 | 2200 | 103.6 | 8.7 | 4.6 | 16.9 | 3.5 | 69.8 |
| 10 | 2400 | 103.5 | 8.7 | 4.7 | 16.9 | 3.4 | 69.9 |
| 11 | 2600 | 94.1 | 7.2 | 4.3 | 16.2 | 3.2 | 63.1 |
| 12 | 2800 | 65.6 | 4.6 | 3.0 | 9.9 | 2.3 | 46.0 |

**Appendix S5. Linear model results between beta diversity and elevation**

| Res. var | Exp. Var | Estimate | Std. Error | t−value | p-value | R^2^ |
| --- | --- | --- | --- | --- | --- | --- |
| Total | Elevation ^2^ | 1.03 × 10^−6^ | 4.06 × 10^−7^ | 2.53 | 0.039 | 0.731 |
|  | Elevation | −4.29 × 10^−3^ | 1.87 × 10^−3^ | −2.30 | 0.055 |  |
|  | Intercept | 4.82 | 2.12 | 2.27 | 0.058 |  |
| Trees | Elevation | 6.67 × 10^−5^ | 2.31 × 10^−4^ | 0.289 | 0.780 | −0.113 |
|  | Intercept | 0.172 | 0.513 | 0.335 | 0.075 |  |
| Shrubs | Elevation | 2.39 × 10^−3^ | 7.53 × 10^−4^ | 3.18 | 0.050 | 0.694 |
|  | Intercept | −5.38 | 1.84 | −2.92 | 0.061 |  |
| Herbs | Elevation ^2^ | 1.50 × 10^−6^ | 4.06 × 10^−7^ | 3.70 | 0.076 | 0.936 |
|  | Elevation | −5.86× 10^−3^ | 1.87× 10^−3^ | −3.14 | 0.017 |  |
|  | Intercept | 5.70 | 2.12 | 2.68 | 0.031 |  |
| Ferns | Elevation ^2^ | 3.62 × 10^−7^ | 4.43 × 10^−22^ | 8.17 × 10^14^ | < 0.01 | 1.000 |
|  | Intercept | −1.42 | 2.29 × 10^−15^ | −6.20× 10^14^ | < 0.01 |  |
| Bryophytes | Elevation ^2^ | 1.26 × 10^−6^ | 6.05 × 10^−7^ | 2.08 | 0.077 | 0.435 |
|  | Elevation | −5.22× 10^−3^ | 2.67× 10^−3^ | −1.96 | 0.091 |  |
|  | Intercept | 5.73 | 2.90 | 1.98 | 0.088 |  |

Res. var = response variable, Exp. var = explanatory variable, Std. error = standard error, R^2^ = adjusted R squared

**Appendix S6. Linear model results between the dominance of functional types and elevation**

| Res. var | Exp. Var | Estimate | Std. Error | t-value | p-value | R^2^ |
| --- | --- | --- | --- | --- | --- | --- |
| Eve. trees | Elevation ^2^ | −8.69 × 10^−7^ | 2.99 × 10^−7^ | −2.91 | 0.017 | 0.646 |
|  | Elevation | 3.68 × 10^−3^ | 1.38 × 10^−3^ | 2.67 | 0.026 |  |
|  | Intercept | −3.51 | 1.56 | −2.25 | 0.026 |  |
| Eve. shrubs | Elevation ^2^ | 1.94 × 10^−6^ | 8.05 × 10^−7^ | 2.41 | 0.052 | 0.527 |
|  | Elevation | −8.33 × 10^−3^ | 3.68 × 10^−3^ | −2.26 | 0.064 |  |
|  | Intercept | 8.87 | 4.12 | 2.15 | 0.075 |  |
| Graminoids | Elevation ^2^ | 9.69 × 10^−7^ | 3.10 × 10^−7^ | 3.13 | 0.012 | 0.568 |
|  | Elevation | −4.22 × 10^−3^ | 1.43 × 10^−3^ | −2.96 | 0.016 |  |
|  | Intercept | 4.66 | 1.62 | 2.88 | 0.018 |  |
| Liverworts | Elevation ^2^ | −5.35 × 10^−7^ | 3.71 × 10^−7^ | −1.44 | 0.183 | 0.227 |
|  | Elevation | 2.27 × 10^−3^ | 1.71 × 10^−3^ | 1.33 | 0.217 |  |
|  | Intercept | −2.08 | 1.94 | −1.07 | 0.312 |  |

Res. var = response variable, Exp. var = explanatory variable, Std. error = standard error, R^2^ = adjusted R squared, Eve. trees = evergreen trees, Eve. shrubs = evergreen shrubs

**Appendix S7. Linear model results between elevational ranges and elevation**

| Res. var | Exp. var | Estimate | Std. Error | t-value | P | R^2^ |
| --- | --- | --- | --- | --- | --- | --- |
| Total | Elevation | −0.230 | 7.20 × 10^−2^ | −3.19 | < 0.01 | 0.456 |
|  | Intercept | 2.43 × 10^3^ | 1.67 × 10^−2^ | 14.5 | < 0.01 |  |
| Trees | Elevation | −0.206 | 8.56 × 10^−2^ | −2.41 | 0.037 | 0.303 |
|  | Intercept | 2.60 × 10^−3^ | 1.99 × 10^2^ | 13.1 | < 0.01 |  |
| Shrubs | Elevation ^2^ | −2.91 × 10^−3^ | 8.01 × 10^−4^ | −3.63 | 0.011 | 0.715 |
|  | Elevation | 12.6 | 3.67 | 3.43 | 0.014 |  |
|  | Intercept | −1.09 × 10^4^ | 4.11 × 10^3^ | −2.66 | 0.037 |  |
| Herbs | Elevation ^2^ | −1.56 × 10^−3^ | 1.05 × 10^−3^ | −1.48 | 0.017 | 0.161 |
|  | Elevation | 6.75 | 4.85 | 1.39 | 0.197 |  |
|  | Intercept | −5.09 × 10^3^ | 5.49 × 10^3^ | −92.8 | 0.378 |  |
| Ferns | Elevation ^2^ | −2.72 × 10^−3^ | 3.96 × 10^−4^ | −6.88 | < 0.01 | 0.883 |
|  | Elevation | 11.7 | 1.71 | 6.84 | < 0.01 |  |
|  | Intercept | −9.90 × 10^3^ | 1.82 × 10^3^ | −5.46 | < 0.01 |  |
| Bryophytes | Elevation ^2^ | 9.58 × 10^−4^ | 3.27 × 10^−4^ | 2.93 | 0.017 | 0.374 |
|  | Elevation | −4.42 | 1.51 | −2.93 | 0.017 |  |
|  | Intercept | 6.84 × 10^3^ | 1.71 × 10^3^ | 4.00 | < 0.01 |  |

Res. var = response variable, Exp. var = explanatory variable, Std. error = standard error, R^2^ = adjusted R squared
